# HBV and HCV Coinfection Associated with Warm-Type Autoimmune Hemolytic Anemia: A Case Report

**DOI:** 10.4274/tjh.2012.0198

**Published:** 2014-09-05

**Authors:** Quan-le Zhang, Li-juan Jia, Jin-biao Zhang, Wei-min Li, Yuan-kai Bo, Jing Li

**Affiliations:** 1 Cangzhou Hospital of Integrated Traditional Chinese and Western Medicine, Department of Internal Medicine, Cangzhou, Hebei, China; 2 Cangzhou Hospital of Integrated Traditional Chinese and Western Medicine, Clinical Laboratory, Cangzhou, Hebei, China

**Keywords:** HBV, HCV, Autoimmune hemolytic anemia

## TO THE EDITOR

The hepatitis virus may play an important role in autoimmune hemolytic anemia (AIHA). We herein report the case of a patient presented to our hospital with hepatitis B virus (HBV) and hepatitis C virus (HCV) coinfection who developed warm-type AIHA. The anemia improved, followed by declined viral levels, after rituximab plus antiviral therapy. To the best of our knowledge, this is the first reported case of HBV and HCV coinfection associated with warm-type AIHA. Written informed consent was obtained from the patient.

**Figure 1:**
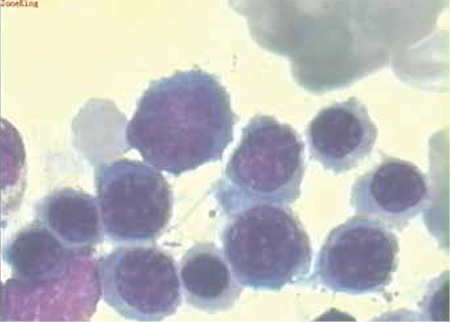
Characteristics of the bone marrow cell morphology: active myelosis and erythropoiesis (stain, Wright; original magnification, 100x).

A 66-year-old woman presented to the department of internal medicine of our hospital on 31 August 2010 with jaundice, inappetence, dizziness, palpitation, and fatigue. A month before admission, the patient complained of fatigue and palpitation, particularly aggravated after exercise, but she did not pay attention to it due to relief of these symptoms at rest. Three weeks later, the patient felt inappetence and dizziness, and her fatigue and palpitation did not go into remission at rest. Her daughter noticed her jaundice at this point and directed her to our hospital. She did not have past medical history of diffuse connective disease, lymphoproliferative disease, transfusion, favism, or allergic contact dermatitis. She had no related social history of cigarette, alcohol, or drug use. Her general physical examination revealed icteric skin and sclerae. Her abdominal examination showed a slightly increased spleen located in the left-upper abdomen. Complete blood count showed severe decrease of red blood cell count (1.39x1012/L), hemoglobin (53 g/L), and platelet count (73x109/L), in addition to significantly increased reticulocyte percentage (19%). Blood biochemistry revealed elevated total bilirubin (6.62 mg/dL), unconjugated bilirubin (4.95 mg/dL), and lactic dehydrogenase (528 U/L), with remarkable decrease of haptoglobin (0.3 g/L). The immunological test displayed positive direct or indirect Coombs results, with warm-type IgG titer against surface antigens of red blood cells elevated. Serologic tests revealed positive HBs-antigen and HCV-antibody, whereas rubella virus, cytomegalovirus, herpes simplex virus, and hepatitis A and E virus were all negative. Subsequently, the quantitative virus analysis showed a significant increase in HBV DNA copies (1.57x107 copies/mL) and HCV RNA copies (1.03x106 copies/mL). Serology testing was negative for factors such as ANA, SMA, anti-dsDNA, anti-RNP, or anti-PNCA (Table 1). Ultrasound of the abdomen showed mild splenomegaly. No imaging characteristic of lymphadenovarix was displayed. The bone marrow aspirate revealed active myelosis and erythropoiesis (Figure 1). Liver biopsy was not performed.

After much consideration, the initial treatment strategy was steroid medication. Oral prednisone at 50 mg/day (1.0 mg/kg) was thus begun. In light of the possibility of aggravated symptoms stemming from single replication of HBV or HCV, or concurrent replication by prednisone, we recommended that the patient meanwhile take oral lamivudine (100 mg/day) and ribavirin (800 mg/day). The patient’s clinical manifestation improved at first. However, it then deteriorated with bilirubin, reticulocyte percentage, and lactic dehydrogenase increasing and red blood cell count, hemoglobin, and haptoglobin decreasing at day 5. Review immunological testing displayed a higher IgG titer, but no significant change of HBV or HCV quantification was demonstrated. Most likely, the treatment failure could be ascribed to the potential steroid resistance in this case. Rituximab was recommended for this patient because of refusal of other immunosuppressants (azathioprine, cyclosporine, or MMF) or intravenous immunoglobulin upon informed consent. An impressive rapid remission of clinical manifestations occurred in the patient after this amended treatment, and laboratory data were improved at day 12 after admission, which allowed us to discharge her to home as an outpatient for further management. Rituximab was not ceased until follow-up day 33, when the anemia disappeared and Coombs testing became negative. At 20 months of follow-up, as of present, antiviral therapy still continued although the loads of the 2 viruses were significantly lower than on initial admission (Table 1).

An association between virus infection and AIHA has long been recognized. A few reports have analyzed the role of hepatitis virus in the initiation or development of AIHA. Tibble et al. [1] presented the first reported case of acute hepatitis A that resulted in acute autoimmune hemolytic anemia. Chiao et al. [2] presented 90 cases of AIHA among 120,908 US veterans with HCV. Yoshioka et al. [3] reported on a 2-year-old Japanese boy diagnosed with warm-type AIHA as an asymptomatic carrier of HBV. However, hepatitis virus coinfection associated with AIHA has rarely been reported. In particular, there is a lack of relevant reports on the relationship between HBV/HCV coinfection and AIHA, in addition to its treatment, although coinfection may have happened via shared routes of transmission in the course of blood exposure in some HBV/HCV-prevailing countries, such as China, Turkey, or Japan. For this patient, we speculated that the occurrence and development of AIHA most likely originated in HBV or HCV infection, for the following reasons. First of all, the patient had no past medical or social history. Second, laboratory data revealed positive HBs-antigen and HCV-antibody, and elevated copies of HBV or HCV, excluding coinfection with other viruses (e.g., HAV, HDV, or HEV). Ultrasound examination did not reveal characteristics of lymphadenovarix. Finally, previous reports have illustrated that HBV or HCV may exert significant roles in the development of AIHA. Nevertheless, it is not yet clear whether HBV or HCV has a synergistic effect in the pathogenesis of AIHA.

The primary management of AIHA is via corticosteroid medication [4,5]. Unfortunately, our patient’s clinical manifestation deteriorated at day 5 after admission. We ascribed the treatment failure to potential steroid resistance in this case. It was necessary to resort to other therapy options. In consideration of more recent data showing that patients with AIHA respond well to rituximab (375 mg/m2) treatment, irrespective of the type of prior treatments that they have received [6,7], rituximab was recommended for our patient. An expedited remission of clinical manifestation occurred in the patient, and laboratory data were improved at day 12 after admission.

It is generally accepted that viruses may be reactivated by immunosuppressants, including corticosteroids. Thus, antiviral therapy was deemed a necessary treatment measure for HBV-infected patients in the course of immunosuppressive therapy, as evidenced by many related studies that revealed that anti-HBV therapy can reduce the high risk of virus reactivation, all other factors being equal. In addition, on the basis of the above-mentioned assumption that the etiology of the patient is HBV or HCV infection, antiviral therapy, as a therapeutic method that can eliminate or inhibit pathogens, seems to be of great importance. However, there were no associated reports in terms of antiviral therapy in HBV and HCV coinfection. A study by Dufour et al. [8] revealed that important management of AIHA associated with viral infection entailed antiviral therapy. Additionally, the current evidence suggests that ribavirin might be tolerated in treatment of HCV infection in thalassemia major patients [9]. Consequently, lamivudine, together with low-dose ribavirin, was applied to treat our patient. However, pegylated alpha-2a interferon was not used, because pegylated alpha-2a interferon can induce life-threatening autoimmune hemolytic anemia. The index of improved anemia was related to the decrease of virus load by continued antiviral medication after withdrawal of rituximab therapy, which fit our expectations.

In closing, we have herein reported the case of a patient with warm-type AIHA that derived from HBV and HCV coinfection. The anemia improved and viral load rapidly declined after rituximab with antiviral therapy. To the best of our knowledge, this is the first reported case of HBV and HCV coinfection with AIHA. More importantly, it is hoped that the treatment strategy in this case might provide a reference for similar cases.

## CONFLICT OF INTEREST STATEMENT

The authors of this paper have no conflicts of interest, including specific financial interests, relationships, and/ or affiliations relevant to the subject matter or materials included.

## Figures and Tables

**Table 1:**
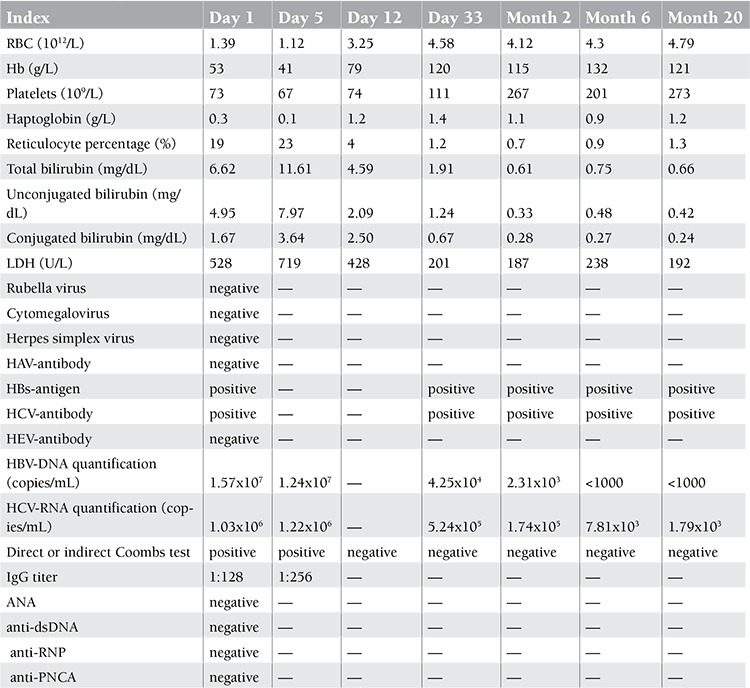
Laboratory data.
